# Sub-Part-Per-Billion Level Sensing of Fentanyl Residues from Wastewater Using Portable Surface-Enhanced Raman Scattering Sensing

**DOI:** 10.3390/bios11100370

**Published:** 2021-10-03

**Authors:** Boxin Zhang, Xingwei Hou, Cheng Zhen, Alan X. Wang

**Affiliations:** School of Electrical Engineering and Computer Science, Oregon State University, Corvallis, OR 97331, USA; zhangbox@oregonstate.edu (B.Z.); houx@oregonstate.edu (X.H.); zhenc@oregonstate.edu (C.Z.)

**Keywords:** fentanyl, surface-enhanced Raman scattering, plasmonic nanoparticles, point of care, public health

## Abstract

Detection of illicit drug residues from wastewater provides a new route toward community-level assessment of drug abuse that is critical to public health. However, traditional chemistry analytical tools such as high-performance liquid chromatography in tandem with mass spectrometry (HPLC-MS) cannot meet the large-scale testing requirement in terms of cost, promptness, and convenience of use. In this article, we demonstrated ultra-sensitive and portable surface-enhanced Raman scattering sensing (SERS) of fentanyl, a synthetic opioid, from sewage water and achieved quantitative analysis through principal component analysis and partial least-squares regression. The SERS substrates adopted in this application were synthesized by in situ growth of silver nanoparticles on diatomaceous earth films, which show ultra-high sensitivity down to 10 parts per trillion in artificially contaminated tap water in the lab using a commercial portable Raman spectrometer. Based on training data from artificially contaminated tap water, we predicted the fentanyl concentration in the sewage water from a wastewater treatment plant to be 0.8 parts per billion (ppb). As a comparison, the HPLC-MS confirmed the fentanyl concentration was below 1 ppb but failed to provide a specific value of the concentration since the concentration was too low. In addition, we further proved the validity of our SERS sensing technique by comparing SERS results from multiple sewage water treatment plants, and the results are consistent with the public health data from our local health authority. Such SERS sensing technique with ultra-high sensitivity down to sub-ppb level proved its feasibility for point-of-care detection of illicit drugs from sewage water, which is crucial to assess public health.

## 1. Introduction

The prevalence of drug abuse and the epidemic level of opioid misuse urgently calls for a comprehensive approach to assess trends in drug abuse and understand the effectiveness of prevention and treatment programs. However, many current surveillance methods of drug use such as surveys, data on overdose incidence, and crime data have a two-year lag before data are available. The other limitations of widely used data sources include lack of geographic resolution, coverage of the selected population, and exclusion of a large portion of the drug-using community. Compared with traditional surveillance techniques, rapid assessment of drug abuse from municipal wastewater [1,2,3,4,5,6] is a new strategy that can overcome many limitations. For instance, municipal wastewater testing methods are readily scalable and anonymous and can eliminate self-reporting surveys, map drug consumption, and can provide an early warning in the constantly changing landscape of substance abuse for proactive responses. High-performance liquid chromatography in tandem with mass spectrometry (HPLC-MS) [7,8,9,10,11] is the most widely used wastewater sensing technique, which can detect various drugs including opioids in the wastewater stream and can differentiate between drugs that were discarded and drugs that were metabolized. Such extraordinary analytical techniques can be adopted to address practical questions and dynamic monitoring of illicit drug use. Specifically, wastewater analysis can reveal the collective drug habits of communities, providing a more accurate picture than self-reported surveys or traditional methods of drug use statistics. It can also alert communities about raising problems in their earlier stage instead of relying on emergency-room statistics and overdose deaths. However, HPLC-MS is an expensive laboratory chemistry analytical technique, which is ill-suited to large-scale, frequent monitoring of illicit drugs in municipal wastewater. It demands labor-intensive sample collection and preparation, expensive reagents and columns, substantive operator expertise, and multiple resources for analysis and calibration. As a comparison, electrochemistry sensors are cost-effective and easy to operate [12,13,14,15,16]. Nevertheless, these sensors cannot probe fingerprints of illicit drugs and lack the necessary specificity for accurate analysis. In summary, there is an unmet need in sensor technologies to address these technological deficiencies and translate wastewater testing into practical insights.

Surface-enhanced Raman scattering (SERS) spectroscopy has become a powerful optical analytical technique due to its high selectivity and ability to obtain vibrational spectra of target molecules at very low concentrations [17,18,19]. Raman peaks are unique and represent the signatures of the vibrational bonds, and therefore Raman spectroscopy is particularly suitable to detect illicit drugs [20,21,22,23,24,25,26]. Compared with other drug sensing methods, SERS techniques can provide exclusive advantages including ultra-high sensitivity [27,28,29], high specificity [30,31], easy sample preparation, low cost, and rapid testing. Especially, the successful commercialization of portable Raman spectrometers in recent years not only significantly reduces the equipment cost compared with bench-top Raman microscopes but also enables a point-of-care (POC) sensing capability, which is much superior to traditional laboratory analytical techniques.

Our previous publications demonstrate that the diatomaceous earth combined with silver nanoparticles (AgNPs) provides additional SERS enhancement [32,33]. Briefly, diatomaceous earth has naturally formed photonic crystal structures. The two-dimensional periodic pores embedded within the diatomaceous earth provide additional optical field enhancement. In addition, AgNPs bring maximal enhancement as the incident laser wavelength is close to the plasmonic resonance frequency of AgNPs. As a result, the combination of diatomaceous earth and AgNPs contributes to extremely high SERS enhancement factors. In this study, we developed a SERS substrate using in situ growth high-density AgNPs on diatomaceous earth and measured fentanyl residual signals using a portable Raman spectrometer to prove the feasibility of sewage water sensing. We achieved a fentanyl detection limit down to 10 parts per trillion (ppt) in artificially contaminated tap water. Based on chemometric analysis, we formed a prediction model of the fentanyl residue concentration using principal components analysis (PCA) and partial least-squares regression (PLSR). Most importantly, we used the developed SERS sensing and data analysis method to measure the fentanyl residue level in the sewage water from the wastewater treatment plant of the City of Corvallis, which was 0.8 parts per billion (ppb), and the mainstream of the Willamette River in Oregon, which was negligible. As a comparison, Table 1 summarizes the performance metrics of different techniques about sensing fentanyl quantitatively in a practical environment. Our diatomaceous earth SERS substrate with a portable Raman spectrometer showed exceeding performance in terms of sensitivity, sensing time, and cost.

## 2. Materials and Methods

### 2.1. Preparation of the SERS Substrate

Glass slides were cut into 0.5 × 0.5 mm^2^ pieces and covered by as-prepared diatomaceous earth solution demonstrated in Reference [37]. AgNPs were then synthesized into diatomaceous earth using the in situ growth method after the samples were dried at 230 °C for 2 h. Based on Equation (1), the in situ growth was performed by the following steps.

(1) $$Sn^{2+}+2Ag^{+}\to Sn^{4+}+2Ag$$

First, samples were immersed into tin (II) chloride (20 mM) and hydrochloric acid (20 mM) for 5 min to create nucleation sites on the surface and within the pores of diatomaceous earth. Second, samples were immersed into silver nitrate (AgNO_3_) (20 mM) for 5 min, after being washed by deionized (DI) water three times and dried at 60 °C for 5 min. This step helped to grow Ag seeds on the diatomaceous earth surface. Third, in order to obtain a mono-layer of uniform AgNPs with high density, the samples were immersed into the aqueous solution of AgNO_3_ (5 mM) and ascorbic acid (20 mM) (v:v = 8:1) for 5 min. Before drying with nitrogen at room temperature, the impurities were removed by rinsing in DI water and acetone three times. The morphology of the active SERS substrate was characterized using a scanning electron microscope (SEM), and the results are shown in Supplementary Materials (SM) Section 1. The prepared SERS substrates were then baked at 150 °C until they were ready for use. For experiments discussed in Section 3.1 and Section 3.2, we fabricated 10 SERS substrates in a single batch. For the comparison of SERS sensing between different sewage treatment plants in Section 3.3, we fabricated 4 SERS substrates in another single batch with the same processing parameters and controlled the variance from the substrate material.

### 2.2. Fentanyl SERS Measurement

**Figure 1:**
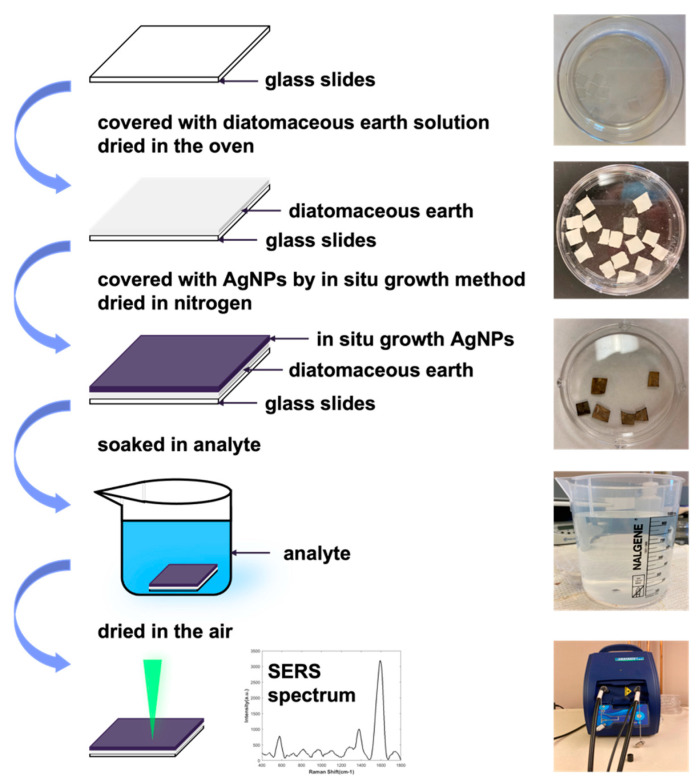
Fabrication process of the SERS substrate using in situ growth of AgNPs on diatomaceous earth. The lowest figure illustrates the characteristic SERS spectra of fentanyl obtained by a portable Raman spectrometer.

Standard fentanyl solution was purchased from Sigma-Aldrich. Tap water was artificially contaminated with standard fentanyl solution at 1 part per million (ppm), 100 ppb, 10 ppb, 1 ppb, 100 ppt, and 10 ppt by magnetic stirring at 700 rpm to achieve homogeneity. One sewage sample was first collected from the outlet of the sewage treatment plant in Corvallis, Benton County, state of Oregon. Its reference sample (mainstream river water) was collected from Willamette River mainstream. For the comparison between different sewage treatment plants, we collected another four samples from the Clackamas, Multnomah, Marion, and Benton Counties, respectively. For each solution sample, we used one SERS substrate. For each substrate, 25 points were measured. The strategy of selecting the 25 points was demonstrated in Supplementary Materials Section 2. The entire detection process was illustrated in Figure 1 with characteristic SERS spectra on the bottom. Active SERS substrates were used to detect the fentanyl concentration in artificially contaminated tap water, sewage water, and mainstream river water. SERS substrates were soaked in the analytes for 24 h and then dried under room temperature before measuring the SERS spectrum with a portable Raman spectrometer (BWS465-5328) equipped with a shaft (BAC102-532) which has a flat quartz window. All samples were excited by a 532 nm laser source with acquisition time of 60 s to collect the SERS spectra in the wavenumber range of 400 to 1800 cm^−1^. The laser power and the integral time were set at 20 mW and 60 s, respectively. The SERS spectra were obtained based on the average of two scanning results.

### 2.3. Multivariate Analysis

We employed the BEADS method to conduct baseline estimation and denoising sparsity [38]. After that, the baseline correction method was used to smooth data. Mean values of those data points and estimate of the analyte concentration were calculated. Multivariant chemometric analysis methods based on PCA and PLSR were then used to extract the features of the SERS spectrum and to establish the relationship between the features and different fentanyl concentrations. First, the contaminated tap water samples were analyzed based on the characteristic peak of fentanyl, which forms a relationship between the peak height and the concentration level. Second, the PCA method was performed based on those data sets to identify the maximum variations between each data set. The first three principal components (PCs) were selected as the major parameters. Finally, PLSR was applied to predict the concentration of target samples.

## 3. Results and Discussion

### 3.1. Fentanyl SERS Detection in Artificially Contaminated Tap Water

**Figure 2:**
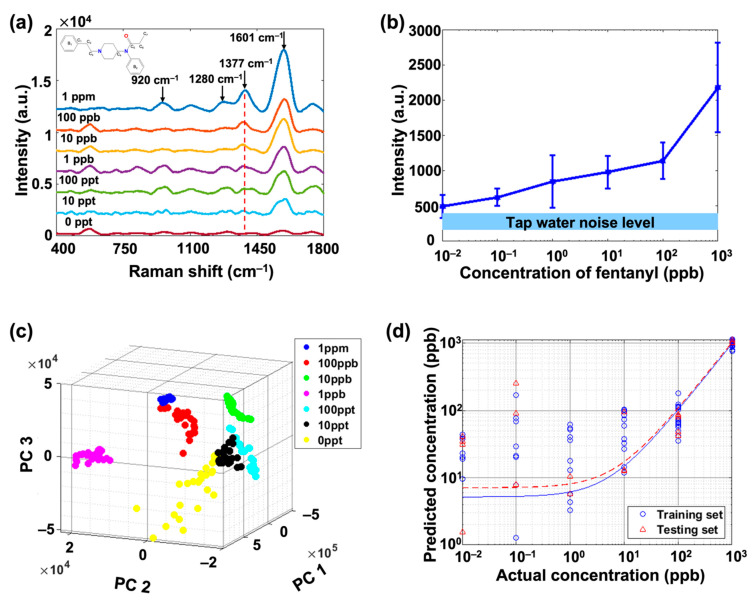
(**a**) SERS spectra of artificially contaminated tap water with fentanyl concentrations from 0 ppt to 1 ppm; (**b**) the intensity of the representative peak at 1377 cm^−1^ with respect to fentanyl concentrations; (**c**) the plot of PCA results for all measured SERS spectra; and (**d**) the PLSR analysis results.

In order to characterize the sensitivity of the POC sensing technique, active SERS substrates were first used to detect fentanyl in a set of artificially contaminated tap water samples with different fentanyl concentrations, as mentioned in the experimental section. The tap water with fentanyl concentrated from 1 ppm down to 10 ppt was measured, and the results are plotted in Figure 2a. As a reference, we provided a SERS spectrum of fentanyl in deionized water in Supplementary Materials Section 3. The labeled SERS peaks were the characteristic peaks of fentanyl. A detailed peak assignment is shown in Table 2 [39].

In our work, we focused on the peak at 1377 cm^−1^ since it is the most representative peak of fentanyl. The results show that the characteristic peak intensity increased as the concentration of fentanyl increased. For a better illustration, the peak intensity at 1377 cm^−1^ was plotted versus the concentration of fentanyl with error bars to indicate the fluctuation of different data points. As shown in Figure 2b, the characteristic peak intensity had a nonlinear relationship with respect to fentanyl concentrations at low concentrations (below 100 ppb). This nonlinear relationship comes from the Langmuir model which is described in Equation (2) [40,41]. In this expression, $C_{a}$ represents the concentration of the analyte, $I_{max}$ is the maximum value of the SERS intensity, K is the absorption constant, and I is the SERS intensity at the concentration $C_{a}$. In Figure 2b, since the horizontal axis is in logarithm scale and the vertical axis is in linear scale, our experimental results followed the Langmuir model.

(2) $$I=I_{max}\frac{KC_{a}}{1+KC_{a}}$$

In addition, the light blue bar at the bottom indicates the background noise level of tap water, which was obtained from the 0 ppt sample in Figure 2a. As the concentration went down to 10 ppt, there were some data points overlapping with the tap water noise bar, which showed the approximate threshold of concentration beyond which the signal could come from noise. Therefore, the sensitivity of our POC sensing SERS substrate was determined to be 10 ppt and was stable for at least 48 h based on our discussion in Supplementary Materials Section 4.

After SERS sensing, we used PCA as a feature extraction technique on these SERS data. This process was conducted by extracting the PCs of these acquired SERS spectra from 400 to 1800 cm^−1^ to account for the effect of concentration levels varying from 1 ppm to 10 ppt. As shown in Figure 2c, the PCA plot qualitatively demonstrated the separation among different fentanyl concentrations, where each point represented a spectrum. The PC1 was the main principal component and accounted for the majority of the score (99.2%). In the PCA plot, when the fentanyl concentration was low, data clusters were close to each other. As the concentration increased, the distribution of the clusters was more separated. Moreover, the slight overlap between 0.01 ppb (10 ppt) and 0 ppb indicated that the sensitivity of the SERS substrate was around 10 ppt, which matches our estimation from Figure 2b. Finally, the PC data were fed into a PLSR algorithm, and a 5-fold cross-validation was performed. In our work, 80% of the data were randomly selected to train the model and the rest of them were used for testing. As illustrated in Figure 2d, the trained model received average R^2^ values of 0.973 and 0.9473 for the training and testing data sets, respectively. The high R^2^ values indicated the strong capability of PLSR to quantify fentanyl concentrations in tap water with high accuracy, especially for a high concentration.

### 3.2. Fentanyl SERS Detection in Sewage Water from the City of Corvallis

**Figure 3:**
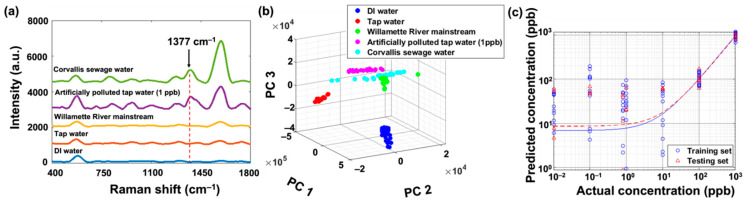
The analytical results of SERS sensing: (**a**) the representative spectra of sewage water and other samples; (**b**) PCA results; and (**c**) PLSR analysis results.

Using the established training model, we measured the fentanyl concentrations in sewage water and mainstream Willamette River water. In Figure 3a, we show the smoothed SERS spectra of DI water, tap water, artificially contaminated tap water with 1 ppb fentanyl, Willamette River mainstream water, and sewage water from the wastewater treatment plant of the City of Corvallis. We found no fentanyl in DI water, tap water, and Willamette River mainstream water as no characteristic peak showed up. As a comparison, sewage water and artificially contaminated tap water shared similar features with a fentanyl characteristic peak at 1377 cm^−1^. By looking into the peak intensity at 1377 cm^−1^, we found that the sewage water was mostly close to 1 ppb. Moreover, we also detected other chemicals from the sewage water. For example, the SERS peak located at 840 cm^−1^ represents pyroborate vibrations, which is not the chemical bond of fentanyl [42]. However, these chemicals did not induce obvious interference with our SERS measurements.

To be more precise, we fitted the raw sewage water data set from the City of Corvallis to the previous PCA-PLSR model. Figure 3b shows the features extracted from the experimentally measured data set. Three clusters represented the Willamette River mainstream, sewage water, and tap water with 1 ppb fentanyl, respectively. The clusters were close to each other as they shared many similar features. Interestingly, the cluster of the Willamette River mainstream is close to the wastewater although we cannot visually see the similarity in Figure 3a. It is possible that the Willamette River may contain a very low level of fentanyl or chemicals with similar SERS spectra. Moreover, the cluster of artificially contaminated tap water with 1 ppb fentanyl close to the wastewater set indicates a similarity in concentration. By introducing these features into the PLSR model as shown in Figure 3c, the predicted concentration in sewage water was 800 ppt with an R^2^ value of 0.9451.

We also tried to measure the concentration of fentanyl in the wastewater using HPLC-MS, which is one of the most popular techniques for quantitative chemical analysis. The results shown in Supplementary Materials Section 5 confirmed the existence of fentanyl in the wastewater. However, the HPLC-MS was not able to provide an exact value of fentanyl concentration, but a range below 1 ppb was estimated. Because the concentration was below the lower limit of quantitation (LLOQ) of fentanyl at 30 ppb using HPLC-MS [34], such results are expected. As a comparison, our SERS technique, together with the PLSR analysis, not only identified fentanyl with ultra-high sensitivity but also quantified the chemical concentration at the range where the HPLC-MS failed to achieve.

### 3.3. Fentanyl SERS Detection from Different Sewage Treatment Plants

**Figure 4:**
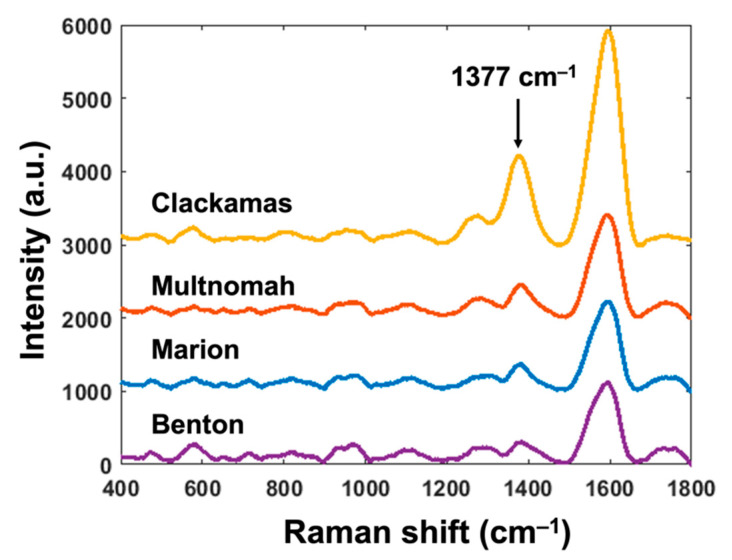
The SERS results from different counties.

In order to further validate our SERS sensing technique, we conducted the same experiments and qualitative analysis for wastewater from sewage plants in different counties, including Benton, whose county seat is the City of Corvallis, Clackamas, Multnomah, and Marion. SERS substrates were fabricated using the same processing parameters as previous experiments. We also followed the same testing and data analysis procedures. The SERS results are shown in Figure 4. All four sewage samples showed the distinguished characteristic peak at 1377 cm^−1^, which confirmed the existence of fentanyl residue. The peak intensity for sewage water samples from Clackamas, Multnomah, Marion, and Benton were 1209.1, 435.6, 359.8, and 294.9, respectively. The intensity of that from Clackamas was much higher than others, indicating a higher concentration of fentanyl residue in the sewage. The peak intensities of sewage samples from Multnomah and Marion are close to each other, indicating similar concentrations of fentanyl residue. The sample from Benton had the lowest peak intensity among the four, representing the lowest level of fentanyl residue concentration in sewage water.

In order to verify our SERS sensing results, we referred to the data of drug use from the Oregon Health Authority (OHA). Based on the most recent data published by the OHA, opioid (non-tramadol) was distributed to 169.075, 138.325, 137.25, and 92.9 people on average per 1000 residents in Clackamas, Multnomah, Marion, and Benton Counties, respectively, from 2019 Quarter 2 to 2020 Quarter 1 [43]. Since fentanyl is one of the most used opioids (non-tramadol), our SERS results are consistent with the public health data from the local health authority. It is worth noting that the sewage water samples we collected from Corvallis, Benton County, in Section 3.3 had lower peak intensity than the sample we collected about a year before as discussed in Section 3.2, although the two samples were both collected from the same location. We believe the difference may come from the COVID-19-related pattern of drug use. Moreover, Benton County has already shown a decreasing trend of opioid (non-tramadol) distribution over years based on the data from the OHA [43], which can explain the lower peak intensity in Figure 4.

## 4. Conclusions

In summary, we developed a rapid POC SERS sensing technique with ultra-high sensitivity to detect fentanyl using a portable Raman spectrometer and PCA-PLSR model. The SERS substrates were fabricated via in situ growth of AgNPs on diatomaceous earth films. We achieved an ultra-high sensitivity down to 10 ppt in artificially contaminated tap water within 2 min in the lab. The PCA-PLSR model predicted the concentration of fentanyl with good R^2^ values for both training and testing data sets. Furthermore, our SERS substrates exhibited a strong sensing capability of fentanyl in sewage water and detected fentanyl in water from a local wastewater treatment plant. The experimental results show that the concentration of fentanyl in the sewage water was about 800 ppt with a high R^2^ value. We also tested the fentanyl concentration through HPLC-MS. The result is consistent with our portable SERS’s but only provided a range below 1 ppb since the concentration was below the LLOQ of HPLC-MS for fentanyl. In addition, we further demonstrated the validity of our SERS sensing technique by comparing SERS results from multiple sewage water treatment plants, and the results are consistent with the public health data from our local health authority. Therefore, we proved that our SERS substrates, when working together with a portable Raman spectrometer and chemometric analysis algorithm, can help an ultra-sensitive sensing technique function at a sub-part-per-billion level to detect the trace level of fentanyl from sewage water, which is crucial to assess public health.

## Figures and Tables

**Table 1:** Different methods of sensing fentanyl quantitatively in practical environment.

Methods	Sensitivity	Equipment Requirement	Estimated Sensing Time	Sensor Cost	Reference
HPLC-MS	30 ppb	HPLC-MS	8 min	High	[34]
Electrochemical	5 ppm	Potentiostat	A few minutes	Medium	[35]
Previous SERS	5 ppb	5lb field-usable Raman spectrometer	5 min	Low	[36]
Our diatomaceous earth SERS	800 ppt	Portable Raman spectrometer	2 min	Low	This work

**Table 2:** Raman shift assignment of the fentanyl SERS spectra.

Raman Shift/ cm^−1^	SERS Peak Assignment
920	C–H asymmetric out-of-plane trigonal bend of B2
1280	C3 C–H twisting
1377	C7 C–H bonds
1601	C–C symmetric stretch of B1

## Data Availability

Not applicable.

## Supplementary Materials

The following are available online at www.mdpi.com/article/10.3390/bios11100370/s1, Figure S1: Morphology characterization of in situ growth AgNPs on diatomaceous earth: (a) optical imaging; (b) scanning electron microscope (SEM) image of the diatomaceous earth on glass slides; (c) SEM image of in situ growth AgNPs on diatomaceous earth; and (d) zoomed-in SEM image of in situ growth AgNPs on diatomaceous earth. Figure S2: The illustration figure of selecting 25 detection points. Figure S3: The SERS spectrum of fentanyl in deionized water, with labeled characteristic peaks. Figure S4: Comparison between the original SERS result and the results after exposing substrate in air for 48 h. Figure S5: HPLC-MS results of Corvallis wastewater with an inserted zoomed-in image of time range from 6.35 to 6.53.
