# Supplementary material for: Sub-Part-Per-Billion Level Sensing of Fentanyl Residues from Wastewater Using Portable Surface-Enhanced Raman Scattering Sensing
Source: Biosensors (Basel). 2021 Oct 3;11(10):370. doi: 10.3390/bios11100370 (PMC8534101; doi:10.3390/bios11100370)
Supplement: Supplementary file 1 [file biosensors-11-00370-s001.zip › biosensors-1367873-supplementary.pdf]

Supporting Information

Sub-Part-Per-Billion Level Sensing of Illicit Drug Residues from Wastewater Using Portable Surface-Enhanced Raman Scattering Sensing

Boxin Zhang[1], Xingwei Hou[1], Cheng Zhen[1] and Alan X. Wang[1,*]

School of Electrical Engineering and Computer Science, Oregon State University, Corvallis, OR 97331, USA; zhangbox@oregonstate.edu (B.Z.); houx@oregonstate.edu (X.H.); zhenc@oregonstate.edu (C.Z.)
* Correspondence: wang@eecs.oregonstate.edu

1. Characterization of in-situ Growth Silver Nanoparticles (AgNPs) on Diatomaceous Earth

Figure S1 shows images of the active SERS substrate captured from an optical microscope and SEM. Figure S1a is a dark-field optical image with 50× magnification, where the bright part is the diatomaceous earth. Figure S1b showed a uniform layer coverage of diatomaceous earth on the glass slide, which indicates a good quality of the SERS substrate. The three-dimensional porous SERS substrate with large surface areas will also increase the adsorption capabilities for fentanyl molecules, leading to enhanced sensitivity. Figure S1c indicates high-density AgNPs covering diatomaceous earth structures using in-situ growth method. Based on Figure S1d, the average diameter of AgNPs and the average distance between adjacent AgNPs were about 30 nm and 25 nm, respectively.

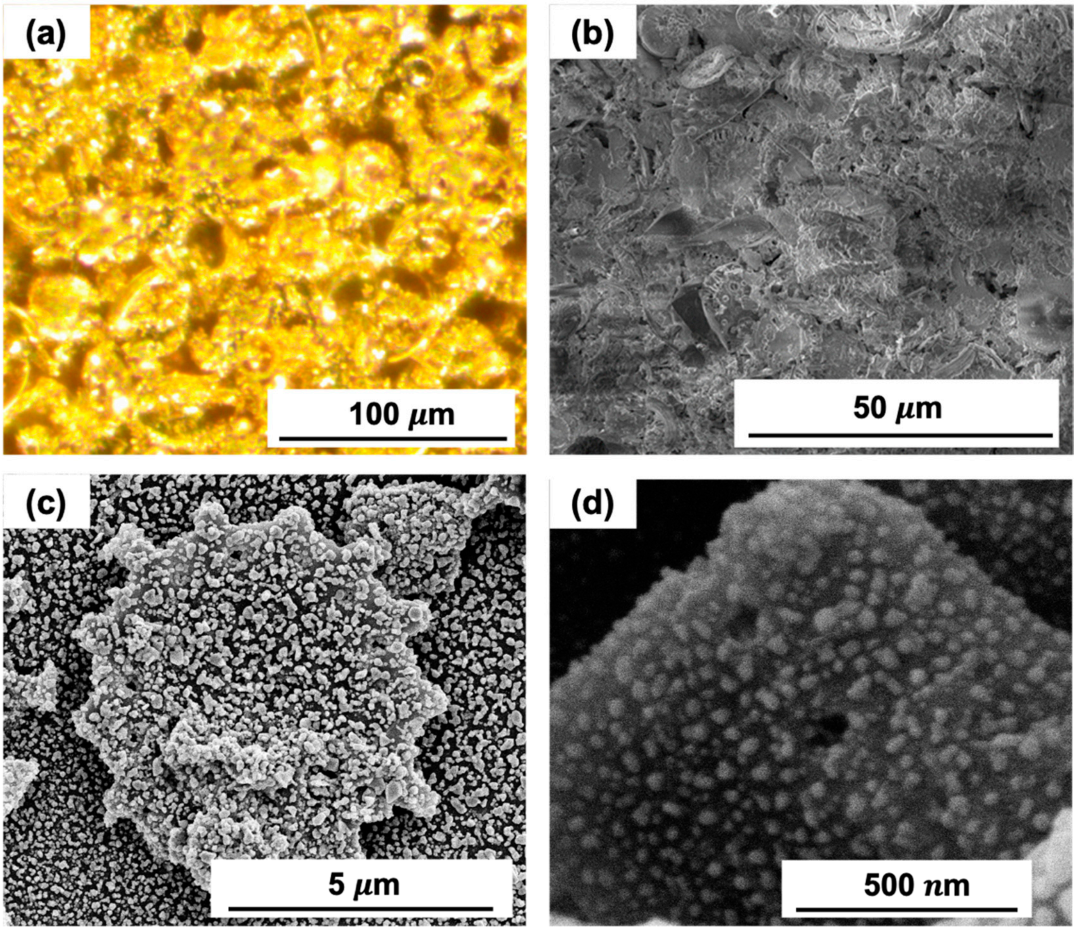

Figure S1. Morphology characterization of in-situ growth AgNPs on diatomaceous earth: **(a)** optical imaging; **(b)** Scanning electron microscope (SEM) image of the diatomaceous earth on glass slides; **(c)** SEM image of in-situ growth AgNPs on diatomaceous earth; and **(d)** zoomed-in SEM image of in-situ growth AgNPs on diatomaceous earth.

2. The illustration of detection points

First, we aligned the Raman laser spot at the center of the SERS substrate. Then, we moved the laser spot within the centered 2 × 2 mm^2 region using a linear transition stage, and measured SERS signals at a 5 × 5 grid so that the 25 points were uniformly distributed within the region. We chose a 2 × 2 mm^2 region inside the 5 × 5 mm^2 sample because the edge of the samples was inevitably scratched by tweezers when we transported and held the substrates.

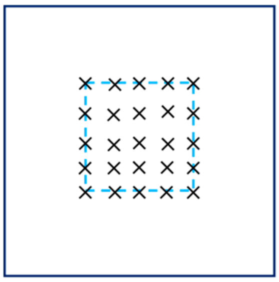

Figure S2: The illustration figure of selecting 25 detection points.

3. The SERS spectrum of fentanyl in deionized water

Figure S3 shows the SERS spectrum of 100 ppb fentanyl in deionized water. The labeled Raman shifts were the characteristic peaks of fentanyl. Table 2 in the manuscript demonstrated the chemical bond assignment of each peak.

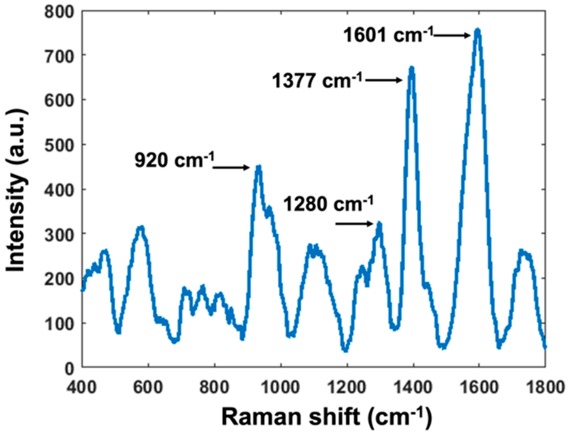

Figure S3: The SERS spectrum of fentanyl in deionized water, with labeled characteristic peaks.

4. The stability of our SERS substrate sensitivity

Figure S4 shows a comparison between the original SERS results and the results after exposing the substrate in air for 48 h. We soaked the substrate into tap water with fentanyl at concentration of 10 ppt and employed the same method as we used in our manuscript for data collection and analysis. The peak intensity at 1377 cm^{-1} was 406 for the original result, and then decreased to 365 after 48 h of exposure. The slightly decreasing intensity indicated that the SERS substrate was stable during a short period. However, long time air exposure increases the oxidation of AgNPs [1]. Therefore, the SERS substrate is not stable over a long period, which is a typical problem for AgNPs based SERS substrates.

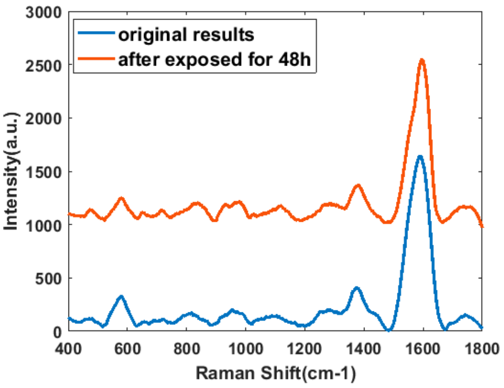

Figure S4: Comparison between the original SERS result and the result after exposing substrate in air for 48 h.

5. High-performance Liquid Chromatography in Tandem with Mass Spectrometry（HPLC-MS）Results of Wastewater

Figure S5 shows the wastewater results from HPLC-MS testing with an inserted zoomed-in spectral analysis of fentanyl ranging from 6.38 to 6.53 min. The shadow area in the inserted imaging is the response of fentanyl in the wastewater. As labeled in the figure, the retention time of fentanyl is 6.49 min, which confirmed the existence of fentanyl in the wastewater. However, the signal-to-noise ratio is lower than the quantitative analysis requirements. Based on the standard calibration curve, the concentration of fentanyl is lower than the quantitative analysis limitation of fentanyl (30 ppb).

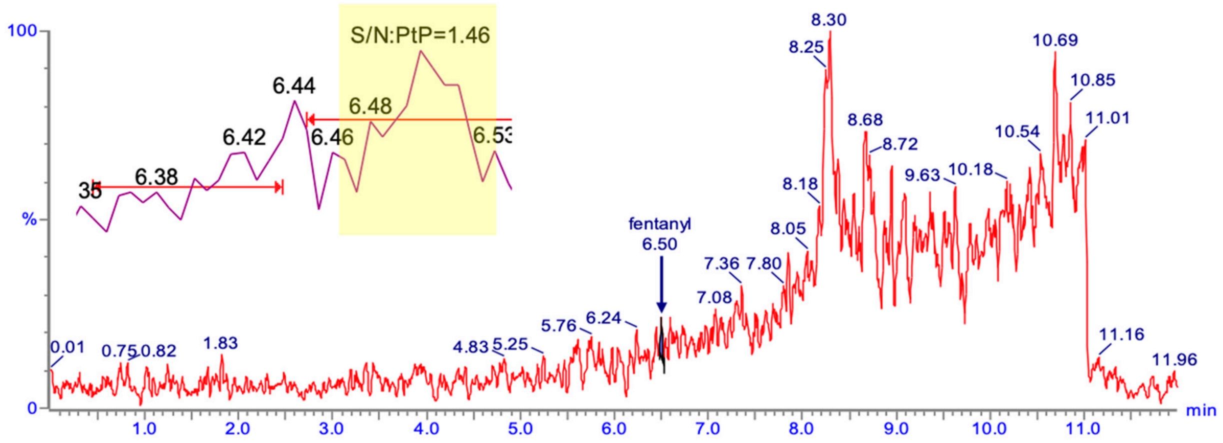

Figure S5. HPLC-MS results of Corvallis wastewater with an insert zoomed-in image of time range from 6.35–6.53.
